# VICO-DR: A Collaborative Virtual Dressing Room for Image Consulting

**DOI:** 10.3390/jimaging9040076

**Published:** 2023-03-26

**Authors:** Gilda Manfredi, Gabriele Gilio, Vincenzo Baldi, Hiba Youssef, Ugo Erra

**Affiliations:** 1 Department of Mathematics, Computer Science and Economy, University of Basilicata, 85100 Potenza, PZ, Italy; 2 Research & Development, Sautech Group (SAUTECH S.R.L.), 84013 Cava de’ Tirreni, SA, Italy

**Keywords:** computer graphics, virtual reality, virtual dressing room, collaborative environment, image consulting

## Abstract

In recent years, extended reality has increasingly been used to enhance the shopping experience for customers. In particular, some virtual dressing room applications have begun to develop, as they allow customers to try on digital clothes and see how they fit. However, recent studies found that the presence of an AI or a real shopping assistant could improve the virtual dressing room experience. In response to this, we have developed a collaborative synchronous virtual dressing room for image consulting that allows customers to try on realistic digital garments chosen by a remotely connected human image consultant. The application has different features for the image consultant and the customer. The image consultant can connect to the application, define a database of garments, select different outfits with different sizes for the customer to try, and communicate with the customer through a single RGB camera system. The customer-side application can visualize the description of the outfit that the avatar is wearing, as well as the virtual shopping cart. The main purpose of the application is to offer an immersive experience, ensured by the presence of a realistic environment, an avatar that resembles the customer, a real-time physically-based cloth simulation algorithm, and a video-chat system.

## 1. Introduction

For many years, fashion has been perceived as a way to express identity, as it provides an indication of social status or lifestyle. Based on the idea that “you are what you wear”, many people search for the best outfit that can send the message they want. Since fashion is counted as a discipline, many of them seek for sector specialists who can recommend the right outfit for every occasion. These specialists, called image consultants, aim to improve the client’s image for social or professional events. According to the London Image Institute, until 1980 the figure of the image consultant was known only among famous people. Nowadays, thanks to the development of a global community, even the general public begins to invest in its appearance to make a good impression. Whether for famous or for regular people, an image consultant must meet and know the customer to provide etiquette, personal hygiene, makeup, hairstyle, and clothing advice. However, in recent years, the world population has faced a serious epidemic that forced people to stay at home. Social distancing norms made people change their habits and search for new ways to do things that are typically performed in person, such as in-store shopping or meeting and collaborating with others. To overcome these limitations, many new immersive and collaborative solutions based on Virtual Reality (VR) and Augmented Reality (AR) have begun to develop. An example is Virtual Shopping (VS), which can be seen as an alternative to in-store shopping and standard e-commerce. With VS, indeed, customers can browse a catalog of items, as any e-commerce store, but can also examine each item in a more immersive way. Among VS solutions we can mention mobile applications which use AR to virtually place 3D furniture models in the real space captured by the phone camera [1,2], virtual showrooms [3], or technologies to virtually try on beauty products using AR [4,5]. Along the same lines of the virtual try-on, Virtual Dressing Rooms (VDRs) [6,7,8] have to also be mentioned. A VDR is an XR application that allows the user to try on digital clothes, see how they fit, and possibly buy the real ones. One of the characteristics shared by VDR solutions is the presence of a camera able to identify the user’s body shape and track its movements in real-time. Some of these solutions also use algorithms to physically simulate garments [9,10] with the aim of enhancing the user’s perception of realism. In light of the above, it is clear that VDRs and other VS techniques can guarantee a better sense of immersion than e-commerce, but may not be enough to ensure a complete shopping experience. Indeed, a study conducted by Xue et al. [11] on a group of people testing a VS platform shows that the presence of an AI or a real Shopping Assistant (SA) could improve the VS experience. This is because a SA can help customers make decisions by providing more information about the product they intend to buy. A virtual SA could be a mobile application that helps customers during in-store shopping by making recommendations for additional products [12,13], or by creating a route in the store for desired products [14]. There are also solutions where both the SA and the store are virtual. For example, Huang et al. [15] proposed a scenario in which a user, with a Head Mounted Display (HMD), enters a VS system. There, a virtual speaking SA helps the user to find and purchase products, redirecting him to the respective showroom. Remaining on the idea of a fully virtual experience, Morotti et al. [16] conducted a study on the potential of a VR–Voice Assistant (VR-VA) environment to empower the user experience. All these solutions revolve around the concept of a non-human SA. If, on the one hand, it allows automation of some processes, such as recommendations, selection, or purchase of a product, on the other hand, it may give users a sense of “unnaturalness”. This can be particularly evident in fashion shopping, where a non-human SA might lack of aesthetic sense compared to a professional figure, such as an image consultant. Furthermore, because fashion is a way to express one’s identity, a non-human SA would not be able to understand the full spectrum of emotions and impressions that an outfit can evoke. For this reason, it would not be able to define an image and a style consistent with the specific personality and peculiarities of the customer. The objective of having a highly immersive virtual fashion shopping solution can be pursued by focusing on the idea of collaborative VR systems. These are characterized by a virtual environment within which multiple users interact with each other and/or with virtual objects [17]. Collaborative systems can be organized by when users collaborate: if they collaborate at different times, the collaboration is asynchronous [18,19]; otherwise, it is synchronous [20]. About the second type of collaboration, there are several applications that use VR for teleconferencing [21], medical data visualization [22], surgical planning [23], training in global engineering collaboration [24], and so on.

Following this idea, we have developed and tested a collaborative synchronous VDR called Virtual Image Consulting Dressing Room (VICO-DR) for image consulting, which provides a unique advantage over related works by introducing a human image consultant who can collaborate with the customer. Using VICO-DR, the customer can try on realistic digital garments, properly chosen by a remotely connected human image consultant. The application provides different functionalities to users based on whether they are an image consultant or a customer. In comparison to related works about VDR applications (see Table 1), VICO-DR has several advantages. Firstly, it uses a common RGB camera system instead of a specialized depth sensor, making it more accessible and easy to set up. Secondly, it can obtain both linear and circular anthropometric measurements in real-time and automatically, which enhances the accuracy of the customer’s high-fidelity 3D model. Thirdly, VICO-DR utilizes Unreal Engine 4.27, a game engine known for generating hyper-realistic environments, and includes a real-time physically-based cloth simulation algorithm, which makes digital garments behave similarly to the real ones, thereby enhancing the sense of realism. In terms of avatar realism, VICO-DR generates a high-fidelity 3D model of the customer, which is a significant improvement over related works that either generate a real image of the user or a less realistic avatar. VICO-DR also has an automatic deformation feature that adjusts the user’s 3D model using real anthropometric measurements, unlike related works that require manual deformation or rely solely on the user’s height and width, resulting in a less precise deformation. The 3D model generated by VICO-DR is able to follow the user’s movements thanks to a body tracking system. Another advantage of VICO-DR is the collaborative image consulting feature, which allows the image consultant to connect to the application and define a database of garments, select different outfits with different sizes for the client to try, and communicate with the client through a single RGB camera system. The image consultant side of the application also includes features such as a customer management system that allows the image consultant to keep track of their customers, their preferences, and previous virtual styling sessions, as well as a virtual shopping cart that allows the image consultant to keep track of the garments selected during the session and provide links or other information to purchase them. Additionally, the customer-side application can visualize the description of the outfit that his avatar is wearing and the virtual shopping cart.

The remainder of this paper is structured as follows: Section 2 provides a brief description of the various technologies used in the development of the system; Section 3 provides an overview of the VICO-DR system architecture; Section 4 describes the setup and the features of the image consultant application, including the server-side and the client-side tasks that the application performs; Section 5 describes the setup and the features of the customer application including the client-side tasks that the application performs; Section 6 includes the results of an experiment conducted on the VICO-DR application to assess the users’ experience; and Section 7 presents the final considerations and future works.

## 2. Background

The proposed approach was developed using Unreal Engine 4.27, Blender version 2.92, the uDraper suite, the HiSQLite3 plugin for UE4, the Agora communication platform, and the MediaPipe framework.

Unreal Engine is a 3D game engine to develop real-time photorealistic and interactive applications. The sense of photorealism is granted by physical simulations, lifelike animations, and sophisticated lighting techniques. Furthermore, UE4 is highly portable and supports multiple platforms, such as PCs, consoles, mobile, AR, and VR devices. Being written in C++, UE4 can be extended via native or third-party libraries.

Blender (https://www.blender.org/download/releases/2-92/ (accessed on 29 January 2023)) is an open-source 3D creation suite that runs on Windows, Linux, and macOS. It offers many tools for modeling, animation, rigging, rendering, texturing, video editing, compositing, and physical simulations. Its functionality can be extended through Python scripts, thanks to the presence of an embedded Python interpreter and several native APIs.

The uDraper (https://udraper.com/ (accessed on 29 January 2023)) suite, developed by the triMirror team, is composed of a third-party software and an UE4 plugin for the modeling and the physical simulation of 3D garments, respectively. With the uDraper modeling software, a user can create a 3D garment starting from a 2D pattern with real measurements. The 3D garment will be simulated within UE4, thanks to the uDraper plugin mentioned above. The physical parameters for the simulation can be set both within the modeling software and the UE4 plugin.

The HiSQLite3 (https://www.unrealengine.com/marketplace/en-US/product/hisqlite3-sqlite3-for-ue4 (accessed on 29 January 2023)) plugin for UE4 is a database management plugin that enables the integration of SQLite3 databases within the UE4 environment. It offers a variety of built-in functions, accessible via C++ or Blueprint, that simplify and automate the CRUD operations of the SQLite3 database. The plugin also allows storing the data in a local SQLite3 database, which can be easily backed up, transferred, and recovered.

The Agora (https://www.agora.io/en/ (accessed on 29 January 2023)) real-time communication platform provides a wide range of services including voice and video calling, live streaming, and interactive broadcasting. The platform is built on WebRTC technology, which enables low-latency, high-quality voice and video communications across different devices and networks. One of the key features of Agora is its ability to support both peer-to-peer (P2P) and client–server (MCU) communication architectures. In a P2P architecture, each participant in a call directly communicates with each other without the need for a centralized server. This results in lower latency and higher-quality voice and video calls, but it also requires more resources from each participant’s device. In contrast, a client-server architecture uses a centralized server to manage and process the media streams, which can reduce the load on the devices and improve the scalability of the service. Agora also provides a wide range of SDKs for different platforms and languages, including C++, Java, Objective-C, and JavaScript. The SDKs can be integrated into various types of applications, including the mobile, web, and desktop. Additionally, Agora provides a cloud-based service, which allows developers to create their own virtual communication channels, and manage the users, media streams, and more. Agora offers a UE4 plugin that provides a set of C++ classes and Blueprints that can be used to initialize and join a call, publish and subscribe to audio and video streams, and manage the call’s settings. The plugin also includes a set of sample projects that demonstrate how to use the SDK in a typical UE4 project.

The MediaPipe (https://google.github.io/mediapipe/ (accessed on 29 January 2023)) open-source and cross-platform framework, developed by Google, provides a set of pre-built solutions for common machine learning tasks, such as object detection, facial landmark detection, and body pose estimation. These solutions are built using a combination of machine learning and computer vision techniques and are optimized for performance and accuracy. Additionally, MediaPipe provides a set of tools for building, deploying, and managing machine learning pipelines, making it a powerful and flexible framework for building multimodal machine learning applications.

## 3. System Architecture Overview

**Figure 1:**
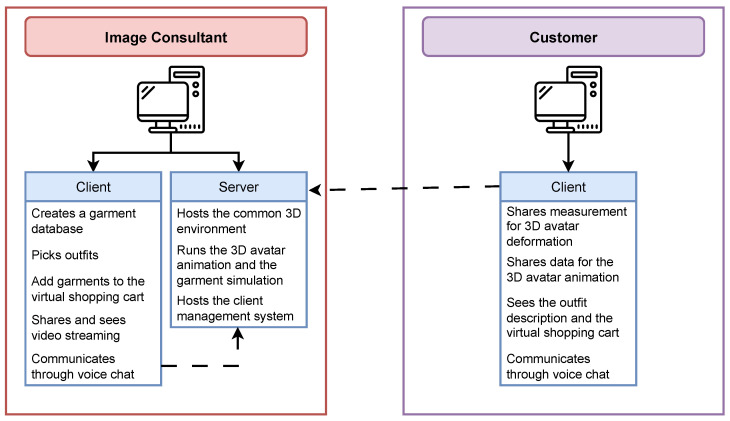
Overview of the VICO-DR system architecture.

VICO-DR allows the image consultant and the customer to collaborate in the same virtual environment. For this reason, the architecture selected for this system is client-server (Figure 1). In particular, the image consultant’s machine is designed to function as both a server and a client, hosting a multiplayer gaming session, accepting connections from remote clients, and actively participating in the game. Conversely, the customer’s machine is only configured to operate as a client. The choice to utilize this particular server mode was made due to its usage as the most prevalent method of cooperative gameplay among a small group of players [25]. The image consultant and the customer share the same virtual environment containing a 3D avatar with the customer’s appearance. The 3D avatar is animated by the movements of the customer and is dressed up in the garments chosen by the image consultant from a database. The garments’ physical simulation and the avatar movements are replicated between the image consultant’s and customer’s machines to keep them synchronized. In addition, the customer and the image consultant can talk to each other through voice chat, and both can see the video-streaming of the image consultant.

## 4. Image Consultant Application

### 4.1. Setup

**Figure 2:**
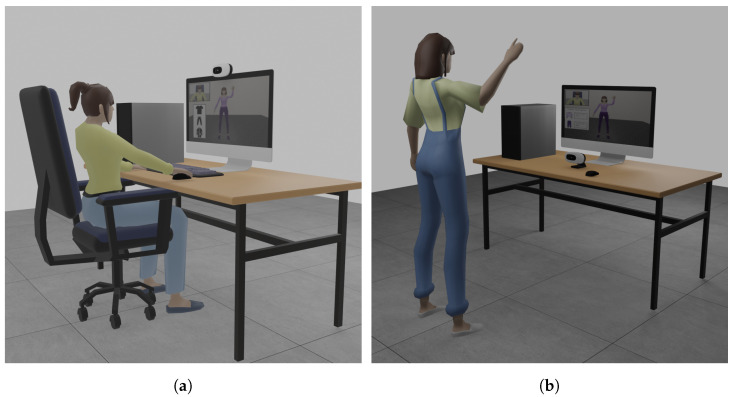
Setup for customer-side (**b**) and image consultant-side (**a**) applications.

The VICO-DR setup for the image consultant-side application (Figure 2a) consists of a computer connected to the internet, a display, and an RGB camera. The image consultant interacts with the application using a mouse and a keyboard. The application was tested using a computer equipped with an AMD Ryzen 7 5800X CPU 3.8 GHz (8 CPU) processor, NVIDIA GeForce RTX 3090 graphic card, (NVIDIA Corporation, Santa Clara, CA, USA) and 32 GB of RAM. The camera used was the Intel^®^RealSense D455 (Intel Corporation, Mountain View, CA, USA).

### 4.2. Server Side

#### 4.2.1. Virtual Environment Configuration

As mentioned above, the image consultant and the customer share the same virtual environment containing a 3D human avatar. In this regard, we present a novel approach for generating highly realistic and detailed 3D avatars suitable for use in the VICO-DR application, but also in other VDR solutions. To achieve this, we adopt the MB-Lab [26] Blender add-on: a comprehensive toolset designed to facilitate the creation and animation of characters for use in 3D animation and gaming applications. This add-on offers a character editor that requires the user to insert the gender, ethnicity, and body measurement to generate a highly realistic and detailed 3D rigged humanoid model. Our approach involves generating a 3D base avatar with realistic body measurements using the MB-Lab character editor, which can be morphed through the Blender “shape keys” [27]. Each shape key corresponds to a specific anthropometric measurement parameter, allowing for the precise customization of the avatar. Our approach also exports the shape keys, commonly referred to as “morph targets”, in an FBX file, along with the mesh and skeleton. The FBX file can be easily imported into a UE4 project. Once imported, these morph targets can be utilized to dynamically deform the avatar during the runtime of the application, when the image consultant initiates a session with a new customer. Since Blender and UE4 do not share the same coordinate system, we developed a Python script that creates the rigged base avatar with its morph targets and makes a conversion between the Blender and the UE4 coordinate systems. This enables the avatar to have the correct size and to be positioned with its feet on the floor and oriented toward the scene camera. Another significant contribution of our study is the incorporation of an additional mesh, referred to as the collision mesh, with a lower Level Of Detail (LOD) into the FBX file, in order to address the performance degradation caused by the garment physics engine when interacting with highly complex meshes. This collision mesh will be used exclusively by the garment physics engine and, as such, will be hidden from the user’s view. It will also include relative morph targets, ensuring that the garments follow character deformations. By reducing the number of vertices in the mesh and consequently the number of collision operations performed by the physics engine, this approach results in improved performance. A more in-depth explanation of the operational principles and functionality of the garment physics engine can be found in Section 4.2.2. At this point, one important consideration about the structure of the avatar skeleton has to be mentioned. As said before, the MB-Lab add-on allows for the creation of a standard skeleton that could be easily imported into the UE4 engine. However, due to the fact that many UE4 plugins are designed to work with the standard mannequin skeleton included in the engine, it was necessary to replace the skeleton generated by MB-Lab with the UE4 mannequin skeleton. For these reasons, we implemented a skeleton replacement operation, which involved adjusting the names and orientations of the bones, as well as eliminating any unnecessary bones not present in the UE4 skeleton. The modified skeleton was then set to the same starting pose as the UE4 mannequin, in order to maintain consistency within the virtual environment. Additionally, in order to keep the user’s focus on the avatar and its outfit, we made the decision to maintain the remaining elements of the virtual environment as minimalistic as possible, thus avoiding any potential distractions.

#### 4.2.2. Garment Physical Simulation

To provide a more realistic experience for the user, we adopt a method for physically simulating virtual garments. Our approach utilizes advanced physics simulations to accurately represent the behavior of the garment as if it were made of real-world materials. Specifically, we use the uDraper plugin, which is designed to physically simulate virtual garments in 3D animation and gaming applications. This method differs from traditional animated virtual garment techniques which rely on keyframe animation [28] to manually define the shape and movement of the garment. The use of physics simulations in our application allows for more realistic and dynamic movement and deformation of the garment, while also providing the ability for the garment to interact with other objects in the scene, such as the avatar’s body. The uDraper plugin employs advanced algorithms that compute the deformation of the virtual garment mesh in each frame, taking into account both internal (e.g., resistance to bending or shear) and external forces (e.g., gravity or collision volumes). A characteristic of the uDraper plugin to take into account is that the virtual garment must be pre-modeled and pre-simulated on the avatar’s collision mesh using the uDraper modeling software. This is because the garment physics calculations are performed based on the vertices of the collision mesh and their order. This constraint seems to be a limitation for the VICO-DR application as the virtual garment must be pre-modeled and pre-simulated, yet the avatar body shape is calculated at runtime and, as such, is not predefined. To address this issue, we have developed a novel solution that involves implementing a base avatar that can be deformed at runtime, as deeply explained in the Section 4.2.1. This allows us to pre-model the garments on the base avatar, whose initial measurements are known beforehand, and subsequently import them into the UE4 project at runtime. By doing this, when a session begins and a garment is picked, its simulation starts in parallel with the avatar morphing in real-time, ensuring that the garments adapt and deform with the avatar’s shape. Once the avatar is deformed, the subsequent garments adapt to its new shape and simulate correctly.

#### 4.2.3. Garment Database Creation

Another important contribution of our approach is the development of a garment database which allows the image consultant to present a wide range of options to the customer and provide personalized recommendations. To create and maintain this database, we used the HiSQLite3 plugin for UE4 (see Section 2). Thanks to this, the image consultant can create a garment database directly from the VICO-DR interface and store it in the image consultant’s machine.

**Figure 3:**
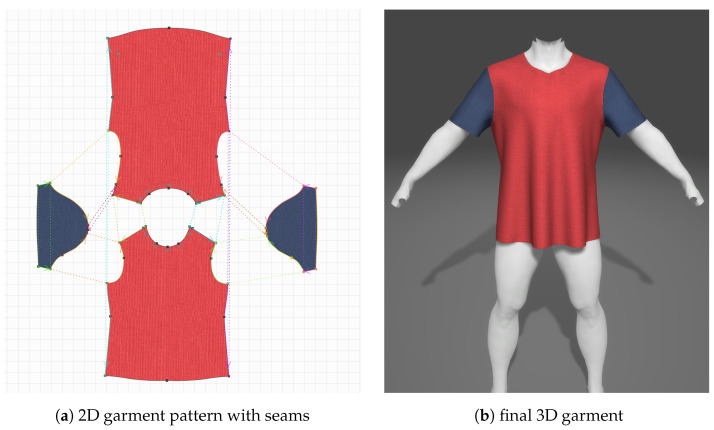
uDraper garment modeling software. The image shows the initial 2D pattern (**a**) and the final 3D garment pre-simulated on the base avatar (**b**).

**Figure 4:**
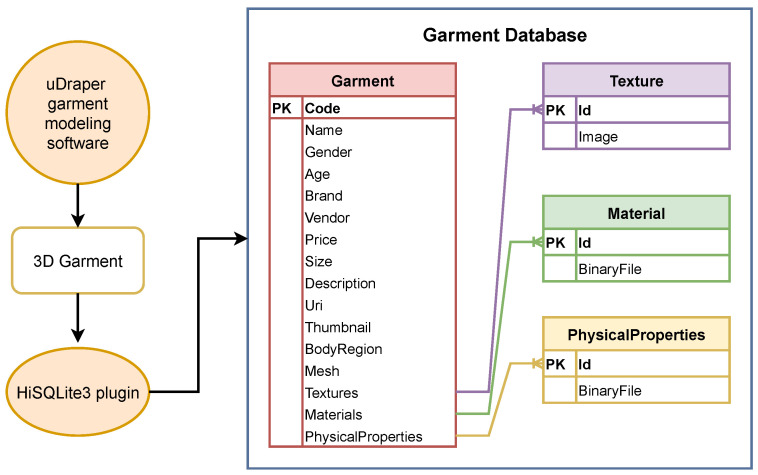
Garment database schema. This Figure shows how the HiSQLite3 plugin creates tables for the 3D Garment exported from the uDraper garment modeling software. The plugin creates four tables: “Garment”, “Texture”, “Material”, and “PhysicalProperties”. The “Garment” table is the main table, and has references to the other three tables related to the garment’s binary files.

To populate the database, the image consultant has to model garments with the uDraper modeling software. The process of creating a virtual garment begins with the design of a 2D pattern (Figure 3a), which can be imported into the uDraper software as an SVG or CAD file or created directly within the software. To enhance the realism of the garment, materials and textures are applied to each section of the pattern, along with physical parameters (weight, damping, friction, etc.) to simulate the behavior of the fabric. Decorative elements such as buttons, zips, and embroidery logos can also be added to the garment. Additionally, the software includes a pattern classification functionality that enables the creation of garments in various sizes. Another important function that is useful for garment-storing in the database is the possibility of adding some metadata, including the garment’s name, the gender and age of the avatar, the brand, the vendor, the code, the description, the URI, the thumbnail, and the region of the body. Once the pattern is established, a character model must be chosen and imported to serve as the reference point for arranging the pieces of the pattern in the 3D scene and for the pre-simulation (Figure 3b). Edges of the pattern must be defined to establish the seams, and then the garment can be simulated and exported as “3D Garment”. This is a folder that contains texture images and other uDraper binary files with information about the garment mesh, including its vertices, edges, and faces, as well as information about the materials and textures used for the garment and the physical properties that define how the garment behaves in simulation. At this point, these files can be loaded into the database. In this regard, we have developed a database schema with tables that contain the garment information taken from the metadata cited before and the information about the size and the price. This information is used within the database to facilitate the organization and search for garments in the application. The table also stores the necessary binary files exported from the uDraper modeling software. Figure 4 shows the schema of the garment database. In particular, for each 3D Garment, the HiSQLite3 plugin creates four tables: “Garment”, “Texture”, “Material”, and “PhysicalProperties”. The “Garment” table is the central table, containing information about each individual garment retrieved from the metadata file. Additionally, it also includes a reference to the other three tables. These store binary files containing information about the textures, materials, and physical properties used in each garment.

#### 4.2.4. Audio–Video Chat System

The VICO-DR application also offers an audio–video chat system, which is not present in other VDR systems. This system enables real-time communication between the image consultant and the customer. To implement this system, we used the Agora plugin for UE4, described in Section 2. As said before, in the VICO-DR application, the image consultant’s machine acts as both a client and a server, so it can join the call as a client and manage the call as a server. In detail, the image consultant machine can use the Agora plugin to create a new communication channel and then invite the customer to join the channel. To do this, the plugin establishes a connection with the Agora server, which assigns a unique channel ID for the call. The channel ID is then shared with the customer’s machine, allowing it to join the call. The Agora plugin allows the image consultant to set up the channel with specific configurations such as the audio codec, the video codec, and the video resolution. Once the customer joins the channel, the image consultant machine can use the Agora plugin to manage the call as a server. This includes monitoring the call status and handling events, such as users joining or leaving the channel.

#### 4.2.5. Customer Management System

In our VICO-DR application, we developed a customer management system that plays a crucial role in maintaining and organizing customer information. This system is designed to store and manage data for each customer, including information from previous sessions, such as the garments that were tried on and the shopping cart for each session. The system was implemented using the HiSQLite3 plugin (see Section 2), which allows for efficient storage and retrieval of customer data. Each customer is assigned a unique identifier, which is used to track their information across multiple sessions. The system also includes various attributes, such as the customer’s name, contact information, and previous session data which can be useful for providing personalized recommendations based on the customer’s previous choices. Our customer management system represents a significant scientific contribution to the field of virtual dressing room systems, as it enables effective customer data management and facilitates data-driven decision-making.

### 4.3. Client Side

**Figure 5:**
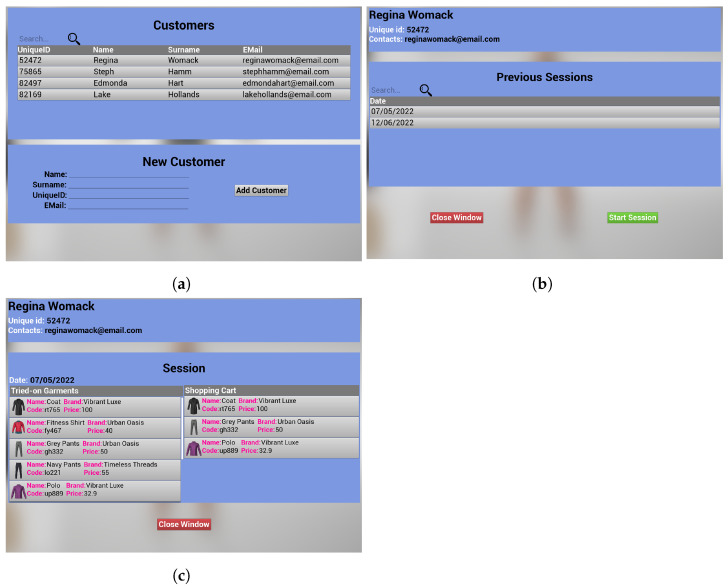
First section of the image consultant application interface, where (**a**) shows the GUI with the customer’s list and the fields to insert a new customer; (**b**) shows the GUI with the previous sessions of a specific customer and the button to start a new session; and (**c**) shows the tried-on garments and the shopping cart of a specific session.

**Figure 6:**
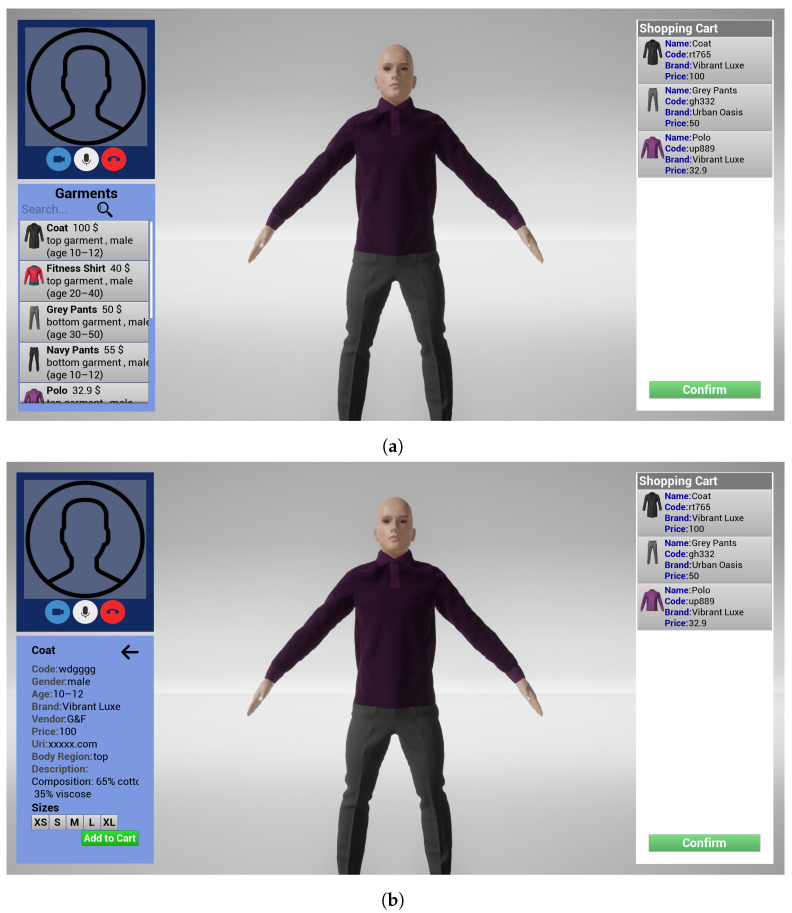
Image consultant VDR interface. The top-left side of (**a**,**b**) shows the video-chat system panel; the right side of (**a**,**b**) shows the shopping cart; the central part of (**a**,**b**) shows the VDR; the bottom-left side of (**a**) shows the garment selection area; and the bottom-left side of (**b**) shows the details of a garment selected in the previous panel.

The client-side application of the image consultant is designed to provide a user-friendly interface for the image consultant to interact with the VDR. The interface is divided into several sections, each serving a specific purpose. The first section contains the customer list, which displays a comprehensive list of all customers that have interacted with the image consultant. The image consultant can add a new customer within the relative panel. Each customer is identified by a unique identifier, and additional information such as the customer’s name and contact details are also displayed (See Figure 5a). By selecting an individual customer from the list, the image consultant can access a detailed view of the customer’s interactions with the VICO-DR system, including a list of previous sessions, which are presented chronologically (See Figure 5b). From this window, the image consultant can also start a new session. By selecting a session entry in the list, the image consultant can analyze all the relevant information about the garments that were tried on during the session and the shopping cart, including details such as the garment’s name, brand, and price (See Figure 5c). This functionality allows the image consultant to easily track and review their interactions with each customer, and to provide more personalized recommendations and assistance during future interactions. The second section (bottom-left panel of Figure 6a) is the garment selection area, where the image consultant can browse and select garments for the customer’s avatar to try on. This section includes various filters and search options to facilitate the process of finding the desired garment. The garments are presented as thumbnails with their name, price, and other relevant information such as gender, age, and region of the body. The image consultant can also filter the garments by brand, vendor, and other attributes, as well as search for specific garments using a search bar. Additionally, the consultant can access the garment’s detailed information (bottom-left panel of Figure 6b), including a detailed description and a URI. For each garment, the image consultant can select different sizes to see how they fit on the avatar and eventually add them to the shopping cart. The third section is the virtual dressing room (central part of Figure 6a,b), where the image consultant can see the selected garments on the 3D model of the customer and verify if they fit well. This section allows the image consultant to provide personalized recommendations to the customer based on their body shape and preferences. The fourth section is the shopping cart area (left panel of Figure 6a,b), where the image consultant can see all the selected garments to purchase, with their name, size, and price. The area also shows the total price of the cart. This section allows the image consultant to review the garments selected for the customer and confirm them to allow the customer to complete the purchase process. The fifth section (top-left panel of Figure 6a,b) provides a button for the image consultant to initiate the call, and other buttons to control the call settings, such as the microphone and camera settings, muting and unmuting the microphone, switching the video on and off, and ending the call. The section also displays the video of the image consultant.

## 5. Customer Application

### 5.1. Setup

The setup of the VICO-DR customer-side application (Figure 2b) consists of a computer connected to the internet, a display, and an RGB camera. The customer interacts with the application simply by standing in front of the camera at a certain distance. This way, the camera can capture its movement and transmit it to the 3D avatar. The application was tested using a computer equipped with an Intel^®^ Core i7-5930K CPU 3.5 GHz (8 CPU) processor, NVIDIA GeForce GTX 1080 Ti graphic card, (NVIDIA Corporation, Santa Clara, CA, USA) and 16 GB of RAM. The camera used was the Intel^®^ RealSense D455(Intel Corporation, Mountain View, CA, USA).

### 5.2. Client Side

#### 5.2.1. Avatar Customization System

**Figure 7:**
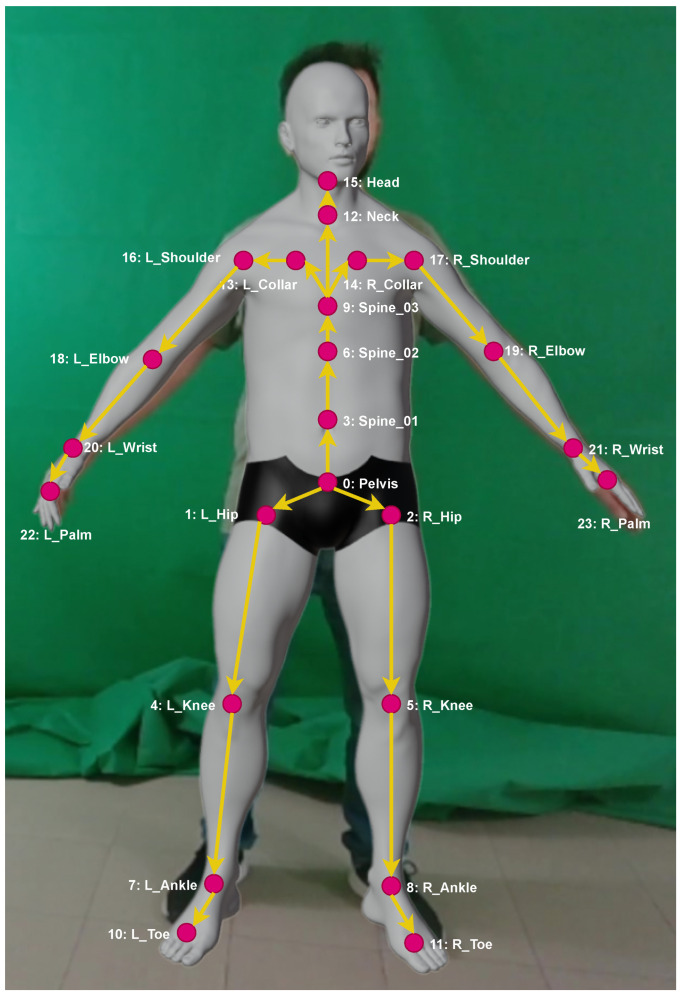
FrankMocap 3D human mesh with the joints’ hierarchy.

**Figure 8:**
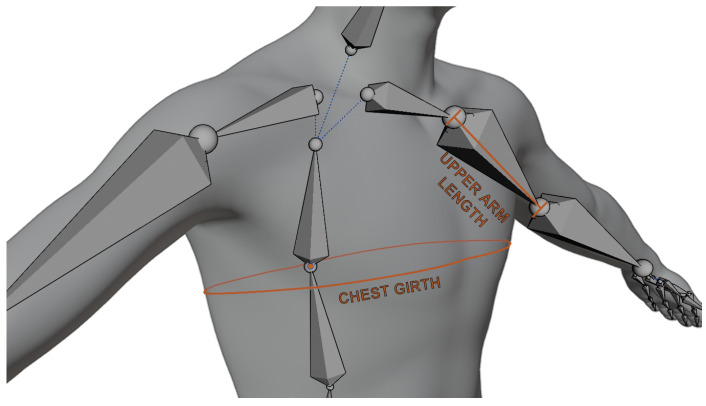
Illustration of linear and circular measurements: upper arm length (linear) and chest girth (circular).

The Avatar Customization System is an integral component of the VICO-DR customer application, allowing customers to personalize their virtual avatars to match their own physical characteristics. Upon starting a session, the Avatar Customization System initiates the calculation of the customer’s anthropometric measurements using advanced computer vision techniques, implemented through the FrankMocap [29,30] framework. This process involves the analysis of 2D images of the customer, captured through an RGB camera. The FrankMocap framework utilizes deep-learning models, specifically trained to reconstruct in real-time a human 3D mesh of the person pictured in the image and a 3D skeleton for the mesh, with a set of body joints (see Figure 7). For the calculation of the anthropometric measurement, we developed a Python algorithm, called Anthropometric Measurement Calculation (AMC), which takes as input the 2D image taken by the RGB camera of the customer, and reconstructs its 3D mesh and the corresponding skeleton. We classified the anthropometric measurements as linear measurements (e.g., height, arm, leg length) and circular measurements (e.g., waist, neck, hips, arm, leg girths). AMC calculates the linear measurements directly on the skeleton by measuring the distance between the relevant joints. For the circular measurements, AMC uses FrankMocap’s body joints as landmarks that indicate the position of a measurement on the skeleton. These positions are used to detect a set of points on the 3D mesh, which are then used to calculate the circular measurements. An example of a linear and a circular measurement can be found in Figure 8. All the measurements are then used inside the VICO-DR application to morph the 3D avatar of the customer. In addition to calculating measurements and deforming the character, the Avatar Customization System allows for storing these measurements for future sessions with the same customer.

To ensure precise anthropometric measurement calculation, the Avatar Customization System requires customers to follow certain guidelines. Firstly, customers must be at the correct distance from the camera, which is typically around 2 m. This distance ensures that the entire body is captured in the image and allows for accurate measurement of body proportions. Secondly, the lighting conditions of the environment must be suitable for image capture, with sufficient lighting to ensure that the image is clear and well-defined. Poor lighting can result in inaccurate measurements and an incomplete 3D mesh. Finally, customers must wear form-fitting clothing that allows for the accurate capture of body contours and proportions. Loose or baggy clothing can result in inaccurate measurements and an incomplete 3D mesh. To ensure that customers are aware of these guidelines, the Avatar Customization System includes clear instructions and prompts throughout the customization process. Additionally, the system includes real-time feedback to customers during the image capture process, alerting them to any potential issues with lighting, distance, or clothing that may affect the accuracy of the measurement. By providing clear guidelines and real-time feedback, we ensure that our Avatar Customization System delivers accurate and reliable anthropometric measurements, resulting in personalized and engaging virtual avatars for our customers.

In conclusion, our contribution includes the development of the AMC algorithm, which is a novel approach to calculating anthropometric measurements from 2D images, and the integration of FrankMocap into the Avatar Customization System, enabling accurate and real-time 3D mesh and skeleton reconstructions from 2D images. Additionally, the Avatar Customization System provides instructions to the customers to ensure accurate measurement capture. These instructions guide the customers on how to position themselves correctly in front of the camera to ensure accurate anthropometric measurements. By providing these instructions, the Avatar Customization System aims to minimize measurement issues that could affect the accuracy of the final avatar.

#### 5.2.2. Motion Tracking System

The Motion Tracking System of the VICO-DR is responsible for mapping the customer’s movements onto its 3D avatar in real time. To accomplish this task, we implemented a UE4 plugin that uses the MediaPipe Pose (https://google.github.io/mediapipe/solutions/pose.html (accessed on 29 January 2023)) estimation system. This machine learning-based solution is able to accurately infer 33 3D landmarks and a background segmentation mask for the entire body from the RGB video frames captured during the session. The integration of the MediaPipe features inside the VICO-DR application is made possible by the fact that MediaPipe supports C++ programming language. In detail, when the collaborative session starts, after the customer’s avatar morphing, the motion-tracking plugin uses the RGB camera to capture the customer’s movements and applies the MediaPipe body tracking pipeline to estimate the 3D joint positions of the customer’s skeleton. Once the skeleton positions are estimated, the plugin maps these joints onto the corresponding joints of the avatar skeleton, making a series of conversions between the MediaPipe landmarks and the avatar’s bones. Overall, our Motion Tracking System offers a sophisticated and efficient solution for real-time mapping of customer movements to their virtual avatars, enhancing the immersive and interactive experience of the VICO-DR application. Moreover, the advantage of our plugin is that it uses a simple RGB camera, making it more accessible and cost-effective compared to other motion-tracking solutions that require more complex and expensive equipment.

#### 5.2.3. Interface

**Figure 9:**
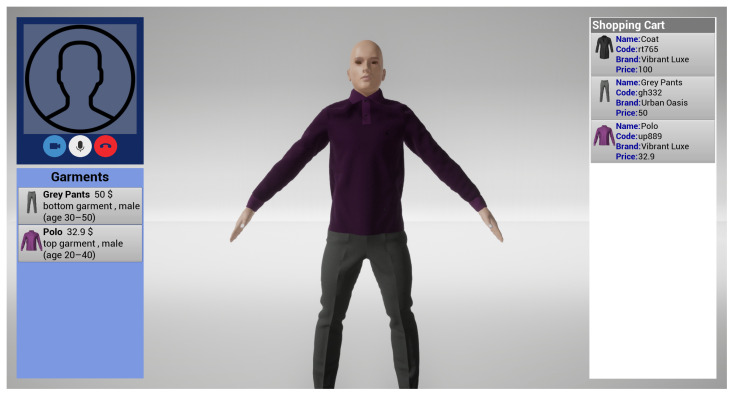
Customer VDR interface. The top-left side shows the video-chat system panel; the right side shows the shopping cart; the central part shows the VDR; the bottom-left side shows the tried-on garments description.

As a complement to the image consultant’s client-side application, the customer’s application also provides a user-friendly interface, which is divided into several sections. The first section comprises two windows. The first one shows the customer information and contains a list of the previous sessions and a button for initiating a connection request to the server hosted by the image consultant’s machine. The second window displays the details of a section selected in the first window. These two windows are equivalent to those present in the image consultant interface, as previously depicted in Figure 5b,c. The information shown in the first section of the customer interface is extracted from the customer management database, which is described in Section 4.2.5. In this way, the customer is able to access only their own session information, as it is identified by a unique code that is assigned upon the establishment of the first session connection. The second section contains a panel that shows information about the outfit that the 3D avatar is trying on at that specific moment (bottom-left panel of Figure 9). The garments are displayed as small images along with their associated attributes, such as name, cost, and demographic information such as gender, age, and body region. The third section is the virtual dressing room (central part of Figure 9), which is shared with the image consultant. This section contains a 3D avatar that closely resembles the customer and mimics its movements in real-time. This allows the customer to visualize how the garments chosen by the image consultant fit on its virtual representation. The fourth section is the shopping cart area (right panel of Figure 9), which is also shared with the image consultant. This section displays a list of the garments that have been selected for purchase, including information such as name, brand, size, and price. The customer is able to review the selected garment and see where to purchase them, as well as view the total cost of the items in the cart. The fifth section of the interface (top-left panel of Figure 9) offers various controls for managing the call settings, including options for adjusting microphone and camera settings, muting or unmuting the microphone, turning the video on or off, and terminating the call. Additionally, this section also displays the video feed of the image consultant.

## 6. Experimental Results

To evaluate the effectiveness and usability of VICO-DR, we recruited a total of 10 participants in a range of ages from 18 to 35. Participants were asked to test the application in groups of two people, where one had the role of an image consultant and the other a customer. The pairs were then asked to switch roles, so each participant could gain a comprehensive understanding of the system’s performance. The testing was carried out in a controlled environment where two computers, each equipped with an RGB camera, were connected to a local network. One computer ran the image consultant application, while the other ran the customer application. The testing session was conducted in a quiet room with controlled lighting conditions. The distance between the participant and the camera was fixed to ensure consistent and reproducible results. Additionally, we asked participants to wear tight-fitting clothes to avoid inaccuracies in anthropometric measurements. Furthermore, we provided a brief training session for each participant to familiarize them with the VICO-DR application before starting the test. During the test, participants were asked to perform several tasks designed to simulate real-world scenarios. For the image consultant-side application, these tasks include creating a customer profile, selecting different digital garments, communicating with the remote customer through the video-chat system, and managing outfits in the virtual shopping cart. For the customer-side application, these tasks include connecting to a session, communicating with the remote image consultant through the video-chat system, and making movements to animate the 3D avatar.

### 6.1. Qualitative Analysis

To conduct a qualitative analysis of the VICO-DR application, we submitted to each of the participants three post-experiment questionnaires: a general questionnaire related to the general user’s experience and two questionnaires related to the users’ experience with the image consultant and the customer-side applications, respectively. The general questionnaire is characterized by seven different questions:

**G-Q1**  *The VICO-DR application was easy to use and navigate.*
**G-Q2**  *The garment simulation algorithm in VICO-DR accurately simulated the movement and behavior of real clothing.*
**G-Q3**  *I found the motion tracking feature in VICO-DR to be accurate and reliable.*
**G-Q4**  *The motion tracking feature in VICO-DR helped me to visualize how different clothes would move and fit on the 3D avatar.*
**G-Q5**  *The anthropometric measurements algorithm creates a realistic 3D avatar.*
**G-Q6**  *The shopping cart is useful to take a trace of/purchase the selected garments.*
**G-Q7**  *I recommend the VICO-DR application to others.*

Each of the seven questions was designed as a five-point Likert scale [31] question, with responses ranging from “Strongly Agree” to “Agree”, “Neutral”, “Disagree”, and “Strongly Disagree”. Participants were asked to select the response that best reflected their experience using the VICO-DR application. The Likert scale is a commonly used method for measuring attitudes and opinions, allowing for the collection of data that can be analyzed and compared. By using this scale, we were able to capture nuanced responses from participants, which provided valuable insight into their perceptions of the application’s performance.

**Figure 10:**
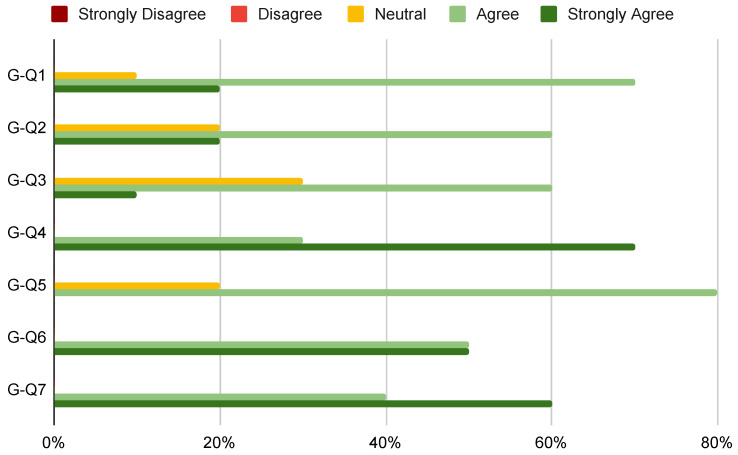
Aswers of the general questionnaire.

The answers given to the general questionnaire (see Figure 10) show that the VICO-DR application was generally well-received by the users. The majority of users found the application to be easy to use and navigate, indicating that the user interface and design were user-friendly. Regarding the garment simulation algorithm, 80% of the users agreed or strongly agreed that it accurately simulated the movement and behavior of real clothing, which is a positive indication of the application’s effectiveness in virtual clothing try-on. The motion tracking feature was generally viewed positively, with 70% of respondents strongly agreeing or agreeing that it helped them visualize how different clothes would fit and move on the 3D avatar. However, 30% of the users were neutral, which suggests that some users may have had mixed experiences with this feature. The anthropometric measurements algorithm, which creates a realistic 3D avatar, was positively received by 80% of users, indicating that it is an effective tool for creating a personalized virtual fitting room experience. The shopping cart feature was also well-received, with 100% of respondents either agreeing or strongly agreeing that it was useful to keep track of or purchase selected garments. Overall, the high percentage of “Agree” and “Strongly Agree” responses for **G-Q7** (60% and 40%, respectively) indicates that users are likely to recommend the VICO-DR application to others, which is a positive indication of the application’s overall user satisfaction.

The other two questionnaires each have additional questions, which are also rated on a five-point Likert scale. The questions of the questionnaire related to the users’ experience with the image consultant-side application are:

**IC-Q1**  *The customer management system in the image consultant application was easy to use and helped me keep track of customers’ information and preferences.*
**IC-Q2**  *Creating a new customer profile was easy to do.*
**IC-Q3**  *Linking a customer to a new session was straightforward.*
**IC-Q4**  *The garment database in the image consultant application is easy to search and navigate.*
**IC-Q5**  *The video-chat system helped me to better understand the customer’s needs and preferences.*

The questions of the questionnaire related to the users’ experience with the customer-side application are:

**C-Q1**  *I found the list of my previous sessions to be useful in remembering the outfits I tried on.*
**C-Q2**  *The 3D avatar accurately represents my body shape and proportions.*
**C-Q3**  *The 3D avatar accurately follows my movements.*
**C-Q4**  *The information displayed in the panel about the tried-on outfits was accurate and helpful.*
**C-Q5**  *The video-chat system allowed me to have a productive consultation with my image consultant.*

**Figure 11:**
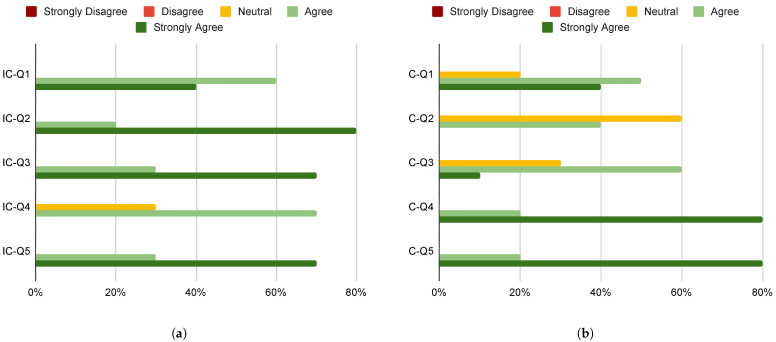
Answers of the image consultant-side (**a**) and the customer-side (**b**) questionnaires.

Based on the responses to the second questionnaire (see Figure 11a), it appears that the image consultant-side application is generally well-received by users. The majority of respondents agreed or strongly agreed that the customer management system was easy to use and helped them keep track of important customer information and preferences. Similarly, most users found creating a new customer profile and linking a customer to a new session to be straightforward. In terms of the garment database, the majority of users agreed or strongly agreed that it was easy to search and navigate. However, there were no respondents who strongly agreed with this statement, indicating that there may be some room for improvement in this area. Finally, the video-chat system was also well-received by users, with the majority agreeing or strongly agreeing that it helped them better understand the customer’s needs and preferences.

Based on the responses to the third questionnaire (see Figure 11b), we can make several observations about the customer’s experience with the customer-side application. For **C-Q1**, while a significant percentage of customers agreed or strongly agreed that the list of previous sessions was useful, there is also a notable proportion who remained neutral. This suggests that while the feature may be helpful for some customers, others may not find it as useful or relevant to their needs. For **C-Q2**, the majority of customers were neutral or agreed that the 3D avatar accurately represents their body shape and proportions. However, a significant proportion of customers remained neutral, which may indicate some level of uncertainty or doubt about the accuracy of the avatar. For **C-Q3**, a majority of customers agreed that the 3D avatar accurately follows their movements, but again, there is a significant proportion of customers who remained neutral. This may suggest that while the technology is generally effective, there may still be some room for improvement or refinement. For **C-Q4**, the vast majority of customers agreed or strongly agreed that the information displayed in the panel about the tried-on outfits was accurate and helpful. This suggests that this feature is a strong point of the application and is generally well-received by customers. For **C-Q5**, a large proportion of customers agreed or strongly agreed that the video-chat system allowed them to have a productive consultation with their image consultant. This is a positive finding, indicating that the video-chat system is an effective means of communication between the customer and the image consultant.

### 6.2. Quantitative Analysis

In order to evaluate the performance of VICO-DR, we conducted several experiments to measure the timing for various operations in the system. We used the setups described in Section 4.1 and Section 5.1.

To measure the time required to start and connect to a session in VICO-DR, we performed 10 trials and recorded the elapsed time from the moment the application was launched to the moment the session was established and ready for use. The average time for the image consultant to create a new session and connect to it was approximately 10 s. Once the image consultant had created a new session, the timing for the customer to connect to the same session was approximately 12 s. After session establishment, the system begins to calculate the customer anthropometric measurements and morph the 3D avatar accordingly. On average, the time required for these two operations ranged from 10 to 15 s. Then, the VICO-DR Motion Tracking System starts to track the customer’s movements in real time. The timing for the image consultant and customer to see the tracked movements depends on the delay in the data transmission from the tracking system to the client–server application. For the image consultant-side application, the delay is in the range of milliseconds, as the image consultant’s machine acts as both the client and server. For the customer-side application, the delay is slightly longer, as the data are transmitted from the tracking system to the image consultant’s machine acting as a server, and then transmitted again to the customer’s machine. The delay may range from a few milliseconds to a few hundred milliseconds, depending on the hardware and network configurations of the customer’s machine and the quality of the internet connection.

At this point, taking into account that the garments are properly organized and categorized, the image consultant is able to select garments within a few seconds using the interface provided by the application. Once the garments have been selected, the physical simulation of garments in the VICO-DR application takes some time to generate. The exact delay depends on the complexity of the garments and the hardware configuration of the image consultant’s machine. For example, if the garments have complex fabric textures and detailed patterns, the simulation may take longer to generate. The delay for the physical simulation of garments may range from a few milliseconds to a few seconds, depending on these factors. Similarly, the delay for the physical simulation of garments for the customer depends on the hardware configuration of their machine and the quality of their internet connection. The physical simulation data need to be transmitted from the server to the customer’s machine for the customer’s machine to render the simulation locally. The delay may range from a few seconds to a few minutes, depending on the complexity of the garments and the hardware and network configurations of the customer’s machine.

The VICO-DR system includes a video-chat system that allows for effective communication between the image consultant and the customer. For the image consultant-side application, the delay is in the range of milliseconds, while for the customer-side application, the delay ranges from a few milliseconds to a few hundred milliseconds.

The VICO-DR system also includes a shopping cart system that allows the image consultant to select garments that the customer will buy. In this case, the timings of operations are almost equal for the image consultant and the customer, with a slight delay of a few milliseconds for the customer’s machine, related to the data transmission through the network.

## 7. Conclusions

In this paper, we have presented a collaborative synchronous VDR solution, called the Virtual Image Consulting Dressing Room (VICO-DR), that aims to improve the shopping experience for customers by introducing a human image consultant. One of the advantages of our approach consists of the use of a combination of UE4 features and a real-time, physically-based garment simulation algorithm to create a highly immersive and realistic virtual environment. The sense of immersion is enhanced by the presence of algorithms that generate a 3D avatar that resembles the customer and that follows its movements in real time. The customer’s avatar can try on digital garments that interact with its mesh in a way similar to reality. Another advantage of the VICO-DR application is the presence of a human image consultant that can use the application to create outfits for customers, track their preferences by checking previous virtual styling sessions, as well as provide links or other information to purchase the garments. All the garments can be created using 2D patterns that are based on existing real-life clothing. The presence of a remotely connected image consultant improves the customers’ shopping experience, giving them a better sense of realism. Our solution is also computationally efficient for the customer side, because all the heavy calculations are made on the server. This can be noticed in the results of the quantitative analysis described in the previous section. The positive impact that the VICO-DR application could have on the image consulting industry has been confirmed by the experiment conducted with 10 participants, which tested the application both as image consultants and as customers. The results of the experiment show that users found the application easy to navigate and use. The 3D avatar accurately represented the user’s body shape and proportions, and the video-chat system allowed for productive consultations between image consultants and customers. The list of previous sessions and the panel displaying information about tried-on outfits were also found to be helpful in the decision-making process. Despite the numerous advantages and benefits of VICO-DR, it is important to acknowledge that there are also limitations to its current implementation. One such limitation is that the MediaPipe body-tracking system, while highly accurate, may experience difficulties in accurately tracking the customer’s movements when they are not directly facing the camera. This can lead to tracking errors and inaccuracies in the virtual try-on experience. In addition, while circular anthropometric measurements are a widely accepted method of measuring body size in VICO-DR, they may be susceptible to slight inaccuracies, particularly in cases where the customer wears bulky clothing. This can result in a less precise virtual representation of the customer’s body, and thus, a less realistic virtual try-on experience. However, this problem can be solved by simply asking the user to wear tight clothes during the measurement process. In light of these limitations, there are several areas for future work. One potential area for future work is the application of deep learning techniques to generate realistic and accurate MetaHuman (https://www.unrealengine.com/en-US/metahuman (accessed on 29 January 2023)) heads using 2D images as input. Additionally, it would be worthwhile to explore the potential of extending the capabilities of the VICO-DR application to the domain of Extended Reality. In this context, fitting digital garments onto a real human body in real time for an Extended Reality VICO-DR application presents a completely different and challenging scenario compared to the traditional method of fitting garments onto a virtual avatar. This scenario requires an accurate and precise measurement of the user’s body dimensions and real-time tracking of their movements to ensure a realistic and seamless experience. One possible solution for achieving this level of accuracy and precision is the use of advanced sensor technologies, such as depth cameras, infrared sensors, or even motion capture suits that can capture the user’s body measurements and movements in real time. This information can then be fed into the VICO-DR application to create a realistic and accurate representation of the user’s body. Another important aspect to consider is the problem of occlusion. Unlike virtual avatars, the customer’s body can occlude parts of the digital garment, making it difficult to achieve a seamless fit. To address this issue, the application would need a sophisticated understanding of the customer’s body and the garment’s structure. One solution could involve using advanced computer vision techniques to segment the customer’s body and the garment separately. By doing so, the application could hide the parts of the garment that are occluded by the customer’s body, giving the impression of a seamless fit. However, this approach would require significant computational power and may not be feasible on lower-end hardware. Additionally, the accuracy of the segmentation algorithm could be affected by factors such as lighting conditions and the complexity of the garment design. In conclusion, VICO-DR represents a significant step forward in virtual reality-based shopping solutions, providing a more comprehensive and personalized experience for customers.

## Figures and Tables

**Table 1:** Comparison table of VICO-DR and other VDR solutions.

	Adikari et al. [6]	Li et al. [7]	Erra et al. [8]	Mok et al. [9]	VICO-DR (Our)
**Game Engine**	Unity	No game engine	Unity	Unity	Unreal Engine 4.27
**Camera System**	Microsoft Kinect V2 (depth sensor)	Common RGB camera	Microsoft Kinect V2 (depth sensor)	Microsoft Kinect (depth sensor)	Common RGB camera
**Anthropometric measurements**	Real (linear and circular)	Not real	Real (only linear)	Real	Real (linear and circular)
**Measurements calculation**	Real-time and automatic	Automatic	Real-time and automatic	Manual	Real-time and automatic
**Avatar Realism**	Real image of user	Not so realistic	Real image of user	Quite realistic	High-fidelity 3D model
**Avatar Deformation**	Manual	Automatic deformation (no anthropometric measurements)	Automatic deformation (only height and width) at runtime	Automatic deformation (by a third-party software)	Automatic deformation at runtime
**Garment Animation**	Keyframe animation	Static fitting without animation	Keyframe animation	Physically-based simulation	Physically-based simulation
**Body Tracking**	Yes	No	Yes	Yes	Yes
**Gesture Recognition**	Yes	No	Yes	Yes	Yes
**Collaborative image consulting**	No	No	No	No	Yes

## Data Availability

Not applicable.
